# Sex determination in beetles: Production of all male progeny by Parental RNAi knockdown of *transformer*

**DOI:** 10.1038/srep00602

**Published:** 2012-08-24

**Authors:** Jayendra Nath Shukla, Subba Reddy Palli

**Affiliations:** 1 Department of Entomology, College of Agriculture, University of Kentucky, Lexington, KY 40546, United States of America

## Abstract

Sex in insects is determined by a cascade of regulators ultimately controlling sex-specific splicing of a transcription factor, Doublesex (Dsx). We recently identified homolog of *dsx* in the red flour beetle, *Tribolium castaneum (Tcdsx).* Here, we report on the identification and characterization of a regulator of *Tcdsx* splicing in *T. castaneum*. Two male-specific and one female-specific isoforms of *T. castaneum transformer* (*Tctra*) were identified. RNA interference-aided knockdown of *Tctra* in pupa or adults caused a change in sex from females to males by diverting the splicing of *Tcdsx* pre-mRNA to male-specific isoform. All the pupa and adults developed from *Tctra* dsRNA injected final instar larvae showed male-specific sexually dimorphic structures. *Tctra* parental RNAi caused an elimination of females from the progeny resulting in production of all male progeny. Transformer parental RNAi could be used to produce all male population for use in pest control though sterile male release methods.

Generation of two sexes (male and female) is an essential and universal phenomenon among animals to reproduce and maintain their existence^1^. In spite of being such a widespread developmental process, different strategies have been adopted by different organisms to accomplish the same goal^2^. Insects are no exception; a myriad of mechanisms are found in insects for their sex determination^3^,^4^,^5^. The homologue of *doublesex* (*dsx*), the bottom most gene of the sex determination cascade in *Drosophila melanogaster*, has been characterized in several insect species belonging to the orders Diptera, Lepidoptera and Hymenoptera^6^. In most of the insect species examined, the pre-mRNA of *dsx* sex-specifically splices to produce one female- and one male-specific RNAs, most likely generating one female- and one male-specific Dsx proteins. The *dsx* pre-mRNAs of *Musca domestica*^7^, *Apis mellifera*^8^, *Bombyx mori*^9^,^10^ and *Aedes aegypti*^11^ are spliced to produce more than two isoforms.

Although the information about the sex-specific splicing regulators of *dsx* pre-mRNA has increased during recent years^3^, not many studies have been performed to characterize the nature of sex-determining signals. An initial signal, at very early stage during the embryonic development, is required to initiate the sex determination cascade; in the absence of this signal the opposite sex develops in a default mode^12^. In *D. melanogaster* the initial sex-determining signal, provided by the X:A ratio^13^ or the dose of X-linked genes (X-Signaling Element, XSE) in females activates the transcription of an autosomal gene *sex-lethal* (*sxl*) from its early promoter^14^,^15^,^16^. Sxl, through an auto regulatory feedback mechanism regulates the sex-specific splicing of its pre-mRNA in turn leading to the production of functional Sxl protein throughout the life^13^,^17^. On the other hand, males which are deprived of the initial pulse of Sxl protein, due to insufficient level of XSEs, accomplish the splicing of *sxl* pre-mRNA in a default mode which produces nonfunctional truncated protein. Sxl protein in females, maintains the splicing of *transformer* (*tra*) pre-mRNA^18^,^19^, leading to the production of functional Tra protein which in association with Tra2 modulates the splicing of *dsx* pre-mRNA in female mode; *dsx* pre-mRNA splices in a default mode in males in the absence of functional Tra protein^20^,^21^. Therefore, in drosophilids XSEs are responsible for producing the initial sex-determining signal by inducing the production of Sxl protein. In medfly, *Ceratitis capitata,* the initial male sex determination signal comes from Y chromosome and in the absence of this signal, in XX embryos the maternal supply of CcTra, the Tra homologue of *C. capitata* initiates female sex determination pathway^22^,^23^. In *A. mellifera*, the initial sex-determining signal is provided, in females, by the heterozygous state of a gene “complementary sex-determining (*csd*) locus”^24^,^25^ but this female-determining signal is maintained by a protein produced by the *feminizer* (*fem*) gene^26^ which is an ortholog of *tra*^25^. *C. capitata*, *Lucilia cuprina* and *Nasonia vitripennis* has been reported to exploit the maternal supply of *tra* as a female-determining signal^27^,^28^,^29^.

Beetles belonging to order Coleoptera of class Insecta, include one fourth of all animal species described and many of them are major pests. Not much is known about the molecular mechanisms of sex determination in this group of insects. Recently, we identified *dsx* homologue in the red flour beetle, *T. castaneum*. *Tcdsx* gene codes for three female-specific and one male-specific isoforms. RNA interference-aided knockdown of *Tcdsx* isoforms revealed isoform-specific functions of *Tcdsx* in *T. castaneum* (Shukla and Palli, in preparation). Here, we report on the identification and characterization of the splicing regulator of *Tcdsx* pre-mRNA, Transformer (*Tc*Tra). Knockdown of *Tctra* induced a change in sex from females to males by diverting the splicing of *Tcdsx* pre-mRNA into male-specific isoform. We have also identified several putative Tra/Tra2 binding sites in the female-specific exon and adjacent introns of *Tcdsx* (Shukla and Palli, in preparation). Also, parental RNAi-aided knockdown of *Tctra* caused an elimination of females from the progeny. *Tctra* parental RNAi could be employed to produce all male populations for use in control of insect pests.

## Results

### Identification of *T. castaneum* Tra/Feminizer

To identify protein responsible for the sex-specific splicing of *Tcdsx* pre-mRNA, we searched (tblastn) the NCBI and the Beetlebase using the known splicing regulators of *dsx* pre-mRNA,Transformer (Tra) protein (P11596) of *Drosophila melanogaster*^20^,^21^, BmPSI (P-element somatic inhibitor) protein of *Bombyx mori* (BAF91871)^30^ and Feminizer protein of *Apis mellifera* (NP_001128300.1)^26^, as a query. Two homologous sequences from *T. castaneum* LOC660887 and LOC100142574 related to PSI and Feminizer respectively were identified. dsRNA specific to these genes were injected in male and female pupae and the knockdown efficiency and splicing status of *Tcdsx* and *Tctra* were assayed using RT-PCR at 5 days after dsRNA injections. Injection of dsRNA of TcPSI did not affect *Tcdsx* splicing pattern; the *Tcdsx* isoforms detected are identical to the isoforms detected in the control insects injected with *malE* dsRNA (data not shown). In contrast, injection of *Tctra* dsRNA affected *Tcdsx* splicing (details are shown in the next section). Based on this RNAi data, we selected *Tctra* as the candidate protein involved in regulation of *Tcdsx* splicing and characterized *Tctra* further.

### Characterization of *Tctra*

**Figure 1:**
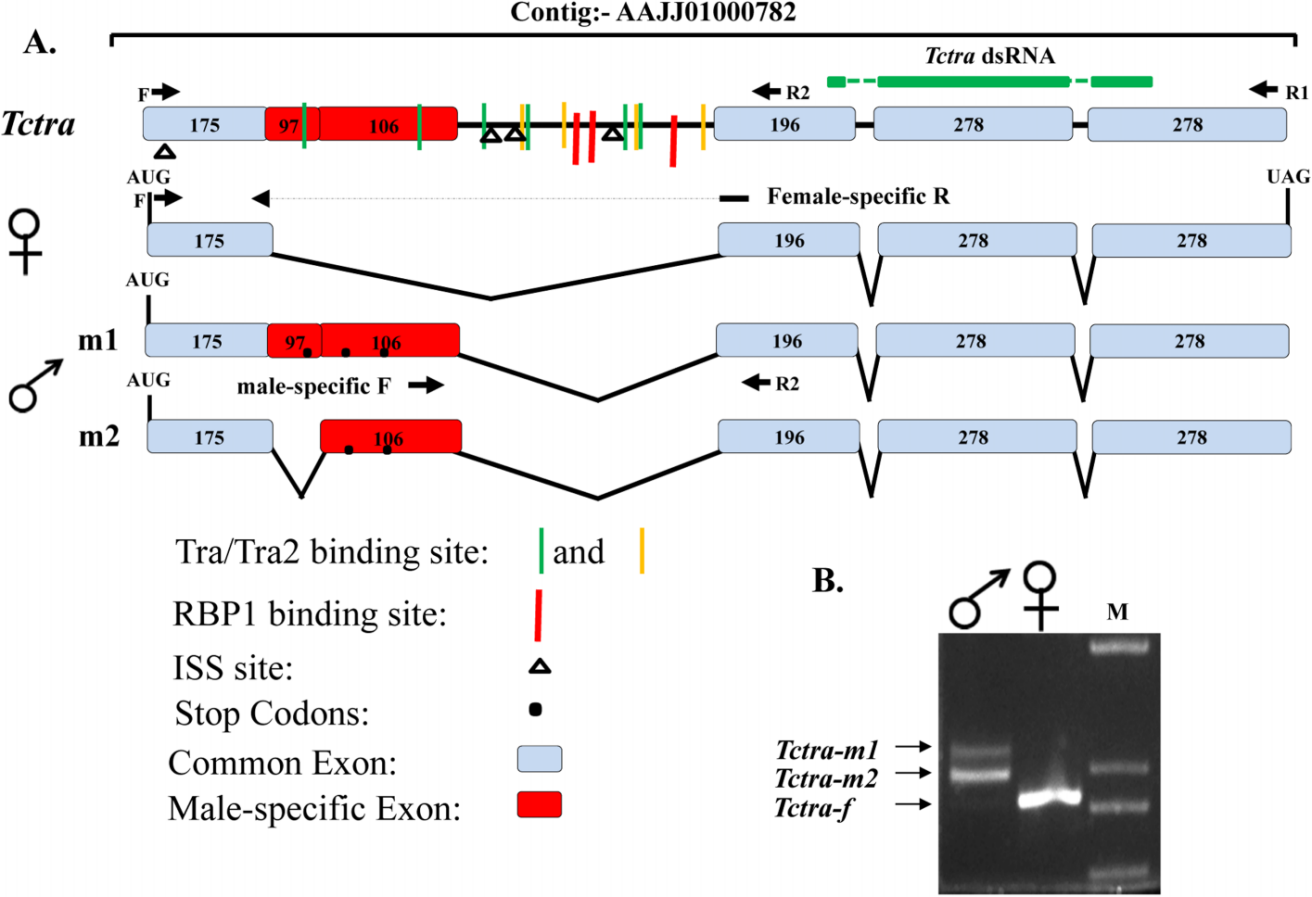
(A) Schematic representation of isoforms of *Tctra* pre-mRNA showing the primer positions and region used for the preparation of dsRNA.Boxes show exons and lines show introns and the numbers in the exons show sizes (bp). Common regions are shown in blue and male-specific regions are shown in red color. Green horizontal bar shows the region used to prepare *Tctra* dsRNA. Black dots represent the presence of stop codons in male-specific exons. Arrows represent the primer locations. Location of putative Tra/Tra2 binding sites are marked using green and yellow vertical lines. RBP1 binding sites are shown using red vertical lines. ISS sites are shown by black triangles. Primers shown as F and female-specific R were used for quantifying female-specific isoform. Primers shown as male-specific F and R2 were used for quantifying male-specific isoforms. (B) Gel picture showing the amplicons generated by PCR using sex-specific c-DNA as templates and F and R1 primers of *Tctra*.

Comparison of deduced amino acid sequence of *Tc*Tra with the related sequences in the databases showed that this is similar to Tra/Fem identified from other insects therefore; *T. castaneum* homologue of Tra/Fem has been named as *T. castaneum* *transformer (Tctra*). RT-PCR was performed using sex-specific cDNAs and primers specific to the ends of *Tctra* ORF (Fig. 1A). A single band of 927 bp and two bands of 1033 and 1130 bp were amplified when female and male cDNAs respectively, were used as templates (Fig. 1B). Sequencing of these sex-specific PCR products and aligning of sequences with the corresponding genomic DNA sequence (AAJJ01000782) identified a complete *Tctra* ORF. Due to the sex-specific alternative splicing of *Tctra* pre-mRNA, male *Tctra* mRNAs contain several in-frame stop codons leading to the production of truncated non-functional protein (Fig. 1A). Putative auto regulation domain, Arg/Ser domain and proline rich region were identified in the deduced amino acid sequence of *Tctra* based on their similarity with those present in Tra/Fem homologs cloned from other insects (Fig. 1S). Sex-specific isoform DNA sequences and the deduced amino acid sequences of *Tctra* have been submitted to the GenBank (Accession no. for *Tctra-f*, *Tctra-m1* and *Tctra-m2* are JQ857102, JQ857104 and JQ857103, respectively). The *transformer 2 (tra2*) gene has been annotated in the *T. castaneum* (XM_963457.2) genome. As reported for other insects, *tra2* of *T. castaneum* (*Tctra2*) expresses in a non sex-specific manner, but we have not performed the detail analysis of *Tctra2*.

**Figure 2:**
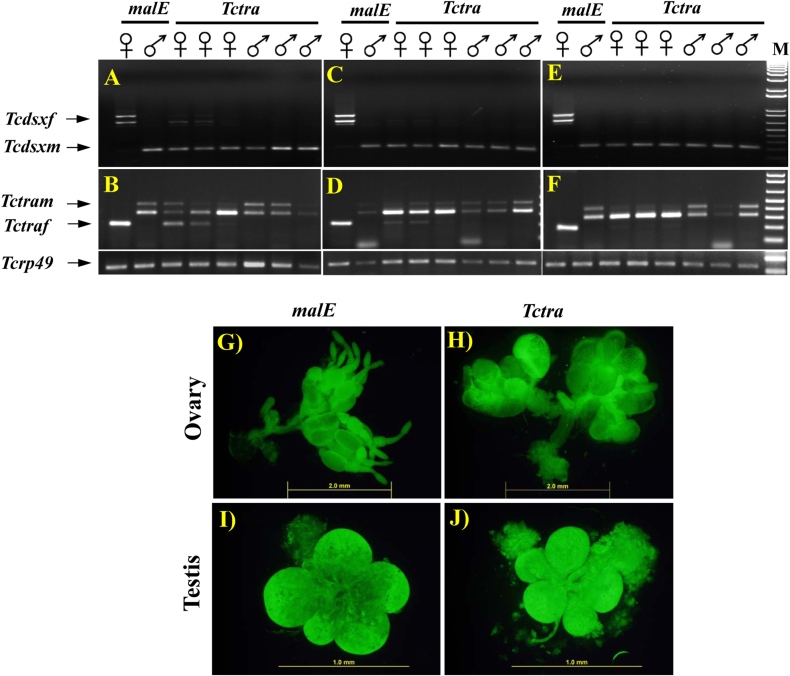
Sex-specific splicing of *Tcdsx* (AC&E) and *Tctra* (BD&F) in *Tctra or malE* dsRNA injected insects. *Tctra* or *malE* dsRNAs were injected into newly emerged (A&B), 48 hr-old adults (C&D) or newly ecdysed pupa (E&F). RNAs were isolated on 5th day after injections and the mRNA of *Tcdsx* and *Tctra* were detected by resolving RT-PCR products on agarose gel. Female and male adults were injected with *Tctra* or *malE* dsRNAs soon after adult emergence. The ovaries (G&H) or testes (I&J) from malE (G&I) or *Tctra* (H&J) dsRNA injected insects were dissected on 7^th^ day after adult emergence, stained with acridine orange and photographed using a fluorescent microscope.

**Figure 3:**
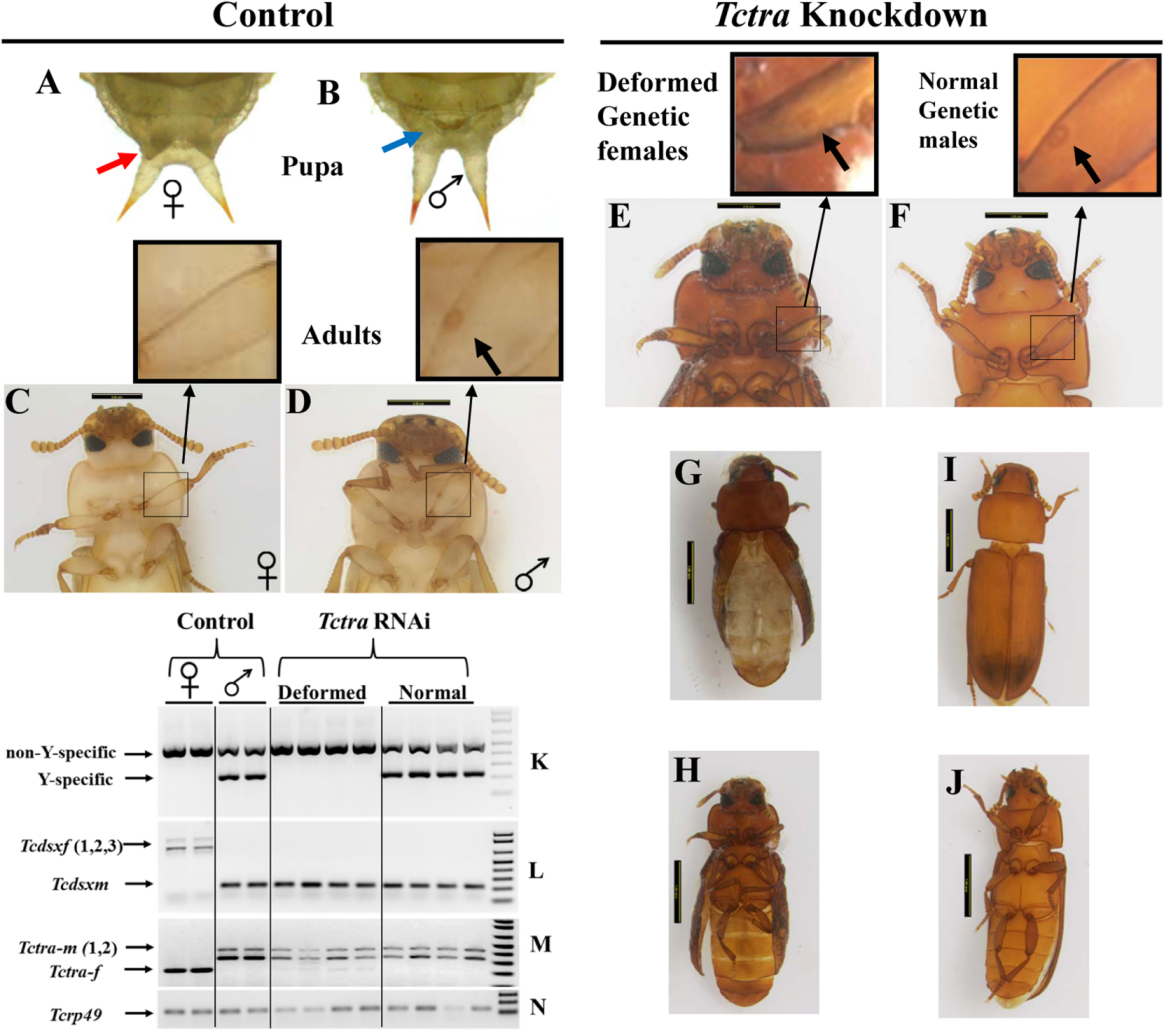
*Tctra* RNAi induced changes in sexual dimorphism during pupal and adult stages. Female (red arrow-A) and male (blue arrow-B) pupae show characteristic papillae. Control females (C) do not show bristle seen on the ventral side of 1^st^ pair of legs in males (D). Enlargements of the region of leg showing the presence (in male) or absence (in female) of bristles are shown in the panel above each picture. All the adults developed from *Tctra* dsRNA injected larvae showed male-specific sex patch (E&F). About 50% of adults emerged from *Tctra* RNAi larvae did not develop properly (G & H dorsal and ventral view, respectively) and died day 2 PAE. The rest of the adults developed normally (I & J dorsal and ventral view, respectively). K. Multiplex PCR with Y-specific and non sex-specific primers^34^ amplifies two bands in males whereas only one band in females. About 50% of adults (deformed) developed from *Tctra* RNAi larvae that showed male phenotype amplified a single band similar to that detected in control females. The rest of the 50% adults showed two bands characteristic of males (normal). L. Genetic males, either from *malE* RNAi or from *Tctra* RNAi larvae showed male-specific *Tcdsx* isoform (*Tcdsxm*). The genetic females developed from *Tctra* RNAi larvae (deformed) also showed *Tcdsxm* isoform in RT–PCR analysis using sex-specific *Tcdsx* primers. M. Genetic males, either from *malE* RNAi or from *Tctra* RNAi larvae showed male-specific *Tctra* (*Tctra-m*). The genetic females (masculinized deformed adults), developed from *Tctra* RNAi larvae showed both *Tctra-m* and *Tctra-f* in RT–PCR analysis using sex-specific *Tctra* primers.

The *tra* genes of several insects have been reported to contain multiple putative Tra/Tra2 binding sites within the male specific exons and the flanking introns^29^,^31^,^32^,^33^. The *tra* of these species also contain sequences similar to intronic splicing suppressor sequence (ISS) and RBP1 binding sites. On the basis of sequence similarity of putative Tra/Tra2 binding sites from these insects, they were grouped into two types, ATCAA type and CAAT type where these nucleotides are found to be 100% conserved (Fig. 2S). The sequences belonging to ATCAA group have five nucleotides upstream and two nucleotides downstream to ATCAA sequence with different degeneracies, probably depending on the insect species (Fig. 2S). The sequences belonging to CAAT group contain four nucleotides upstream and five nucleotides downstream to CAAT sequence with different degeneracy. We searched for the presence of Tra/Tra2 binding sites, ISS sequence (CAAGG/A) and putative RBP1 binding sequences (Type A: DCADCTTTA and Type B: ATCYNNA) in the *Tctra* gene. Ten putative Tra/Tra2 binding sequences, four similar to ATCAA type and six similar to CAAT type were found in the male-specific exons and the adjacent intron sequences (Fig. 1A and Fig. 3S). Three putative RBP1 binding sites ATA(T)A(T)CTTTA, and four ISS sites (Fig. 1A and Fig. 3S) were also detected in *Tctra* DNA. The presence of multiple putative Tra/Tra2 binding sites, ISS sequences and RBP1 binding sites in the male-specific exon and the adjacent intron of *Tctra* suggest the possibility of autoregulation of *Tctra* splicing.

### *Tctra* RNAi experiments

To verify the predicted function of *Tctra* as a splice regulator of *Tcdsx* pre-mRNA, dsRNA targeting *Tctra* was injected into newly ecdysed adults and the total RNA isolated on the 5^th^ day after injections were used to assay *Tcdsx* and *Tctra* mRNAs. In qRT-PCR analysis efficient knockdown of *Tctra* in both female and male insects tested was observed (data not shown). Interestingly, the male-specific *Tcdsx* isoform was detected in all the female insects injected with *Tctra* dsRNA (Fig. 2A). Also, both female- and male-specific isoforms of *Tctra* were detected in all the *Tctra* RNAi females (Fig. 2B). In contrast, all the male insects injected with *Tctra* dsRNA showed no change in the splicing pattern of *Tcdsx* and *Tctra*; only male isoform of *Tcdsx* and *Tctra* were detected (Fig. 2A & 2B). Similarly, male-specific isoform of *Tcdsx* and both male- and female-specific isoforms of *Tctra* were detected in *Tctra* dsRNA injected females and no change in splicing of *Tcdsx* and *Tctra* in males was observed when the *Tctra* dsRNA was injected into 48 hr-old adults (Figs. 2C&D) or newly ecdysed pupae (Figs. 2E&F).

The ovaries dissected from the *Tctra* RNAi adults on the 7^th^ day post-adult ecdysis (PAE) showed significantly different morphology compared to the ovaries dissected from the control females injected with *malE* dsRNA (Figs. 2G&H). The ovaries from RNAi insects showed lobes similar to the lobes present in testes and these ovaries showed no signs of oocyte development (Fig. 2H). Besides, *Tctra* RNAi females mated with normal males did not produce even a single egg (Table 1). *Tctra* RNAi males, on the other hand, developed normal testes (Fig. 2J) similar to the testis observed in males injected with *malE* dsRNA (Fig. 2I). Also, females mated with *Tctra* RNAi males produced equal number of eggs as that of females mated with males injected with *malE* dsRNA (Table 1). These data suggest that *Tctra* is required for production of female-specific isoforms of *Tcdsx* that regulate female reproduction.

### Expression of *Tctra* during larval stage is required for development of pupal and adult sexually dimorphic structures

Pupal and adult stages of *T. castaneum* show sexually dimorphic structures which make it easy to separate males from females, unambiguously. Papillae, two finger-like structures just anterior to the urogomphi, can be used to separate female pupae (Fig. 3A) from the male pupae (Fig. 3B), since the male papillae are much smaller and look like fingertip rather than fingers (http://www.ars.usda.gov/Research/docs.htm?docid=12892). Males (Fig. 3D), during the adult stage show a small patch of short bristles on the inner side of the first pair of legs (1/3 distance from the base) whereas these bristles are absent in the females (Fig. 3C, http://www.ars.usda.gov/Research/docs.htm?docid=12892). In spite of the splicing of *Tcdsx* pre-mRNA to male isoform (*Tcdsxm*, Fig. 2E), splicing of *Tctra* to both male- and female-specific isoforms (Fig. 2F) and the change in the ovaries into testis-ike lobes (Fig. 2H) in female adults developed from the *Tctra* RNAi pupae, no changes in the sexually dimorphic structures was observed during the pupal or adult stages and these structures in *Tctra* RNAi insects are similar to the untreated insects (Fig. 3A–D). To determine whether the development of these sexually dimorphic structures depend on the presence of *Tctra* during larval stages, *Tctra* dsRNA was injected into newly molted 4^th^, 5^th^ and 6^th^ instar larvae. None of the pupa developed from *Tctra* dsRNA injected larvae showed the female-specific papillae and about 50% of the pupae developed from control larvae injected with *malE* dsRNA showed female-specific papillae (Table 2). In addition, all the adults developed from *Tctra* dsRNA injected larvae showed the male-specific bristles (Table 2, Fig. 3E & 3F). About half of the adults developed from *Tctra* dsRNA injected larvae were deformed (Fig. 3G&H) and the other half are normal (Fig. 3I&J).

Multiplex PCR using Y-specific and non sex-specific primers^34^ that amplifies two bands in males and one band in females was employed to identify genetic males and females using the genomic DNA isolated from the *Tctra* dsRNA injected insects as well as two male and two female untreated control insects. Approximately, 50% of adults developed from *Tctra* RNAi larvae were genetic females (since there was no amplification of Y-specific region in genomic PCR, (Fig. 3K) but showed male phenotypes; absence of female papillae during the pupal stage whereas, the presence of male-specific bristles during the adult stage (Fig. 3E). Strikingly, these converted males (genetic females) did not develop normally and showed deformities in development of wing and other appendages (Fig. 3G&H) and died on day 2 PAE. Male-specific isoform of *Tcdsx* (Fig. 3L) and both female- and male-specific isoforms of *Tctra* (Fig. 3M) were detected in these genetic females that showed male phenotypes. The other half of the adults developed from *Tctra* RNAi larvae are genetic males (Fig. 3K), developed similar to the untreated control insects and showed male-specific isoforms of *Tcdsx* (Fig. 3L) and *Tctra* (Fig. 3M). These data showed that expression of *Tctra* during final instar larval stage is a prerequisite for development of pupal and adult sexually dimorphic structures.

### *Tctra* is maternally transferred

**Figure 4:**
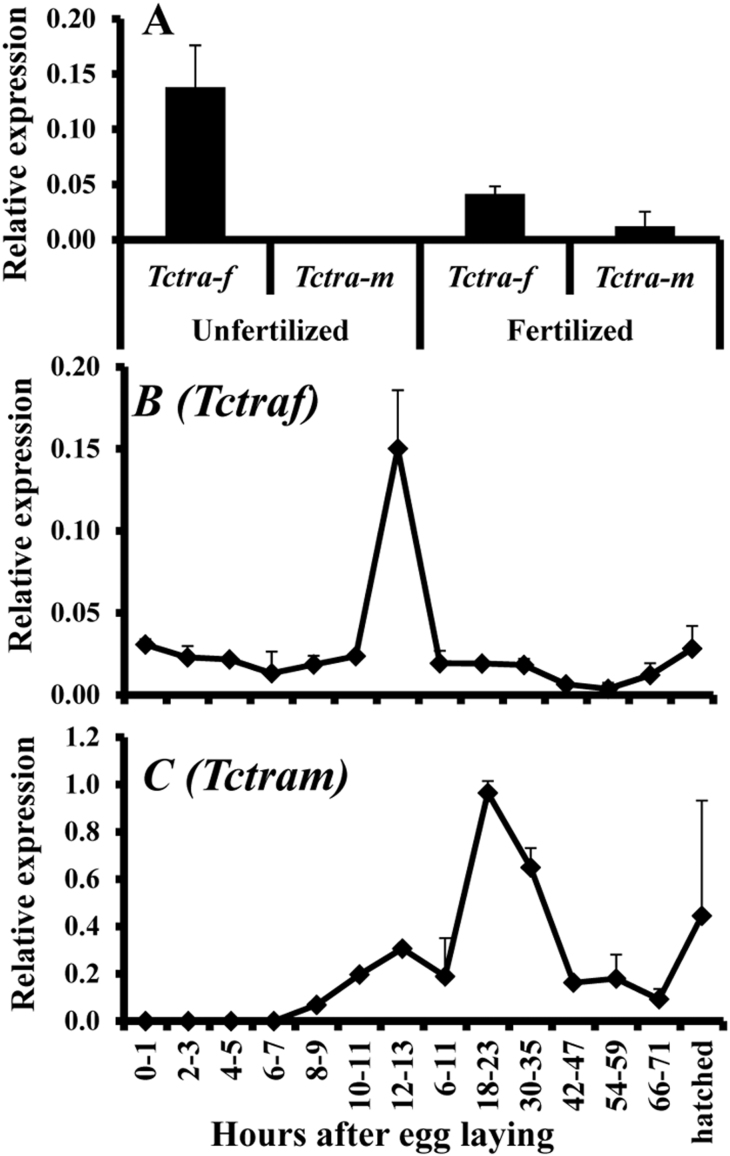
(A) Relative expression of female (*Tctraf*) and male-specific (*Tctram*) isoforms of *Tctra* in unfertilized (0–12 hr after egg laying (unmated females laid fewer eggs therefore, 12 hr collection was necessary to obtain enough eggs) and fertilized eggs (0–5 hr after egg laying).Eggs laid over 0–12 hr period by unmated females and 0–5 hr period by mated females were collected, total RNA was isolated and the mRNA levels of female- and male-specific isoforms of *Tctra* were quantified by qRT-PCR. Higher levels of female-specific *Tctra* mRNA were detected in both fertilized and unfertilized eggs. (B &C) Relative change in the expression of female-specific isoform (B) and male-specific isoform (C) of *Tctra* during the embryonic development. Eggs laid by mated females over an hour period were collected and incubated at 30°C. Samples were collected at 2 hr intervals until 12 hr after egg laying. Similarly, eggs laid by mated females over 5 hr period were collected and incubated at 30°C. Samples were collected at 12 hr intervals until hatching. Total RNA was isolated and the mRNA levels of female- and male-specific isoforms of *Tctra* were quantified by qRT-PCR. A peak of female-specific isoform of *Tctra* is detected at 12–13 hr after egg laying and a peak of male-specific isoform of *Tctra* is detected at 18–23 hr after egg laying.

The expression of sex-specific isoforms of *Tctra* during the embryonic development was analyzed using qRT-PCR. cDNAs prepared from the RNA isolated from the staged embryos were used as templates to perform PCR using primers specific to female or male isoforms of *Tctra*. As shown in Figure 4A, higher levels of female-specific *Tctra* mRNA when compared to the levels of male-specific isoform were detected in both fertilized and unfertilized eggs collected during early stages of embryonic development. Interestingly, the mRNA levels of male-specific isoform of *Tctra* are very low to undetectable in both fertilized and unfertilized eggs during the early stages of embryonic development (Fig. 4A). qRT-PCR analysis of *Tctra* mRNA levels in staged eggs showed that a peak of female-specific isoform of *Tctra* is detected at 12–13 hr after egg laying (Fig. 4B) while a peak of male-specific isoform of *Tctra* is detected at 18–23 hr after egg laying (Fig. 4C). The presence of female-specific isoform of *Tctra* in the unfertilized eggs suggests that this mRNA may be maternally transferred.

### *Tctra* dsRNA injected females produce only male progeny

**Figure 5:**
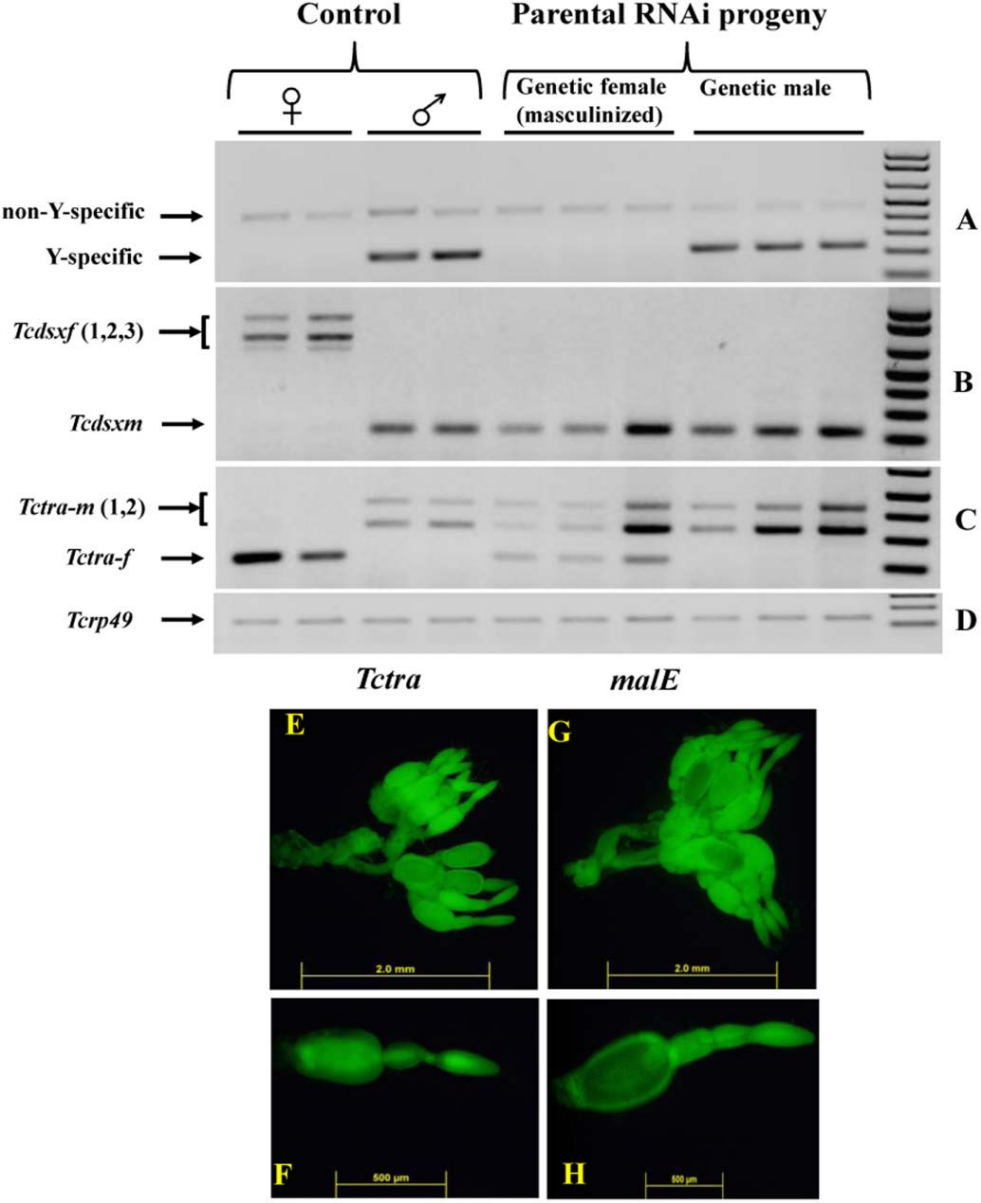
Parental RNAi of *Tctra* affects splicing of both *Tcdsx* and *Tctra* pre-mRNA. (A) Multiplex PCR with Y-specific and non sex-specific primers amplifies two bands in males whereas only one band in females. About 9% of adults developed from eggs laid by females injected with *Tctra* dsRNA showed male phenotype during the pupal stage (the presence of male papillae) and the adult stage (the presence of male-specific bristles) amplified a single band similar to that detected in control females. All the other adults (~91%) were genetic males since two bands (as in control males) were detected. (B) Genetic males developed from eggs laid by either *malE* or *Tctra* dsRNA injected females showed male-specific *Tcdsx* isoform (*Tcdsxm*). The rare genetic females (escapers), developed from *Tctra* dsRNA injected females, also showed *Tcdsxm* isoforms. (C) Genetic males developed from eggs laid by either *malE* or *Tctra* dsRNA injected females showed male-specific *Tctra* (*Tctra-m*). The genetic females (masculinized) developed from eggs laid by *Tctra* RNAi females showed both *Tctram* and *Tctraf* isoforms. (D) Amplification product of *rp49* shown as a control. (E)–(H) The ovaries from *Tctra* (E&F) or *malE* (G&H) dsRNA injected adults were dissected on 20^th^ day after injection of dsRNA, stained with acridine orange and photographed using a fluorescent microscope. F&H show higher magnification image of a single ovariole.

We employed parental RNAi that works well in *T. castaneum*^35^,^36^ to target maternally transferred *Tctra* mRNA. *Tctra* or *malE* dsRNA were injected into female adults on the 5^th^ day PAE; 24 hr after injections the females were mated with un-injected virgin males*.* Five *malE* or *Tctra* dsRNA injected females and five untreated males were placed in separate cups for mating and the larvae hatched from the eggs laid by *malE* or *Tctra* dsRNA injected females were counted on 20^th^ day after initiation of mating. When compared to the *malE* dsRNA injected females, *Tctra* dsRNA injected females produced fewer larvae (Table 3). All the pupae and adults developed from the larvae hatched from the eggs laid by the *Tctra* dsRNA injected females developed into males (evident by the presence of sexually dimorphic structures during pupal and adult stages). When all these males were mated with un-injected virgin females, the number of eggs produced in a week period by each female mated with male developed from *Tctra* RNAi insects is similar to the eggs laid by females mated with normal males (shown in supplementary Table 1S). Analysis of the genetic sex of all the males developed from eggs laid by *Tctra* RNAi females showed that only three out of 34 tested are genetic females and the rest of them are genetic males (Fig. 5A, Table 3). These genetic females and genetic males (along with control females and males) were analyzed for the presence of sex-specific isoform of *Tctra* and *Tcdsx*. Genetic males showed usual male-specific isoforms of *Tcdsx* and *Tctra* (similar to untreated control males) but genetic females developed from eggs laid by *Tctra* RNAi females showed male isoform of *Tcdsx* (Fig. 5B) and both female and male isoforms of *Tctra* (Fig. 5C). The ovaries dissected from female adults injected with *Tctra* dsRNA showed smaller oocytes (Fig. 5E&F) when compared to the ovaries dissected from malE injected adults (Fig. 5G&H). Parental RNAi of *Tctra* showed the requirement of *Tctra* during early stages of embryonic development of XX females.

## Discussion

The order Coleoptera contains the largest group of insects^37^ and the red flour beetle, *T. castaneum* is an excellent model for this group because RNAi works efficiently and systemic and the genome of this insect has been sequenced^38^. We recently identified *dsx* homolog from *T. castaneum* (*Tcdsx*) which codes for three female-specific and one male-specific isoforms. The functions of *Tcdsx* isoforms in sex determination and maintenance were also investigated employing RNAi (Shukla and Palli, in preparation). In this paper, we report the identification and functional characterization of splicing regulator of the *Tcdsx* pre-mRNA, *Tctra*. Searches of *Tribolium* genome sequence using Tra/Fem sequences previously identified in dipteran and hymenopteran insects identified *Tctra*. Further analysis of *Tctra* sequences revealed that the pre-mRNA of *Tctra* is sex-specifically spliced into one female- and two male-specific isoforms. Only female isoform codes for protein containing autoregulation, Arg/Ser and proline rich domains conserved in Tra/Fem proteins identified in other insects (Fig. 1A, Supplementary Fig. 1S^39^). Interestingly, Tra/Tra2 binding sequences, RBP1 binding site and ISS are also present in *Tctra* (Supplementary Figs. 2S&3S).

Knockdown in the expression of gene coding for *Tctra* led to the conversion of genetic females to males. This change is seen in soma (disappearance of female papillae during the pupal stage and appearance of male-specific bristles in *Tctra* knockdown female adults) when *Tctra* dsRNA injections were performed during the larval stages. Changes in germ line tissues were observed when *Tctra* dsRNA was injected during the larval, pupal or adult stages. Therefore, *Tctra* appears to regulate both germ cell and soma sex determination and maintenance, the differences observed could be due to the temporal differences in development of structures studied. Similar case of female-specific regulation of germline and soma, by *feminizer*, has been reported in honey bees^26^.

When parental RNAi was used by injecting *Tctra* dsRNA into adult females to silence the expression of the gene coding for *Tctra* during early embryogenesis, all the larvae hatched from eggs laid by RNAi females were males. The number of eggs laid by *Tctra* RNAi females was significantly lower compared to the number of eggs laid by the *malE* RNAi control females. After *Tctra* dsRNA injections, oogenesis and fertilization may have proceeded in these females in a normal fashion until *Tctra* knockdown took place. Later, after *Tctra* knockdown, both oogenesis and fertilization would have been affected. Consistent with this, we found a reduction in size and arrest in maturation of oocytes in *Tctra* RNAi females compared to the control females at 20 days after injection of dsRNA (Fig. 5G&H). Since *Tctra* dsRNA was injected into 5^th^ day PAE females, the oocytes that matured already^40^,^41^ would have been developed, fertilized and laid by RNAi females. These data show the requirement of TcTra (and TcDsxF) for maintenance of female sex even in the sexually mature adults. Testing the genetic sex of the adults developed from the eggs laid by the RNAi females showed only 3 out of 34 insects to be genetic females (Table. 3). The *Tctra* dsRNA injected into the females may have resulted in a reduction in the amount of *Tctra* mRNA transferred to egg. Alternatively, the *Tctra* dsRNA injected into the females may have been transferred to the eggs along with *Tctra* transcript and the dsRNA cleaved and degraded the maternally supplied *Tctra* transcript. It is also possible that the knockdown in the expression of *Tctra* may have occurred both in the mother as well as in the embryo after transfer of both mRNA and dsRNA. In either case, the end result is the presence of too low levels of *Tctra* during the initiation of sex determination cascade. Interestingly, the three genetic females (in the progenies of *Tctra* parental RNAi parents) were masculinized and produced sperm, successfully mated with females and the mated females produced approximately the same number of eggs as the females mated with normal males.

In *D. melanogaster*, through the process of dosage compensation, the genes in X-chromosome are hyper-activated, in males, by a group of proteins (forming “dosage compensation complex”) equalizing the overall amount of X-linked gene products in females (2X) and males (1X)^42^,^43^,^44^. The dosage compensation complex is not formed in females, deficient in Msl2 (Male-specific Lethal-2) protein (a key component of dosage compensation complex); Sxl protein (present only in females) suppresses the translation of *msl2* mRNA by binding to its UTR sequences^45^. Highly skewed sex ratio in the adults developed from the eggs laid by *Tctra* RNAi females is likely due to the mis-regulation of dosage compensation gene(s). Over expression of X- linked genes in *Tctra* RNAi females may have caused lethality. The presence of *Tcdsxm* isoform was detected in all the 3 masculinized genetic females. These genetic females (converted to functional males due to parental *Tctra* RNAi) would have escaped the zygotic death probably because of the presence of TcTra (protein) above the threshold level to inhibit the activation of dosage compensation pathway (and hence no death of genetically masculinized females) but likely, below the levels required to execute the splicing of *Tcdsx* pre-mRNA into female mode. This argument is supported by the previous work on the *D. melanogaster* recessive mutants for the *virlizer* (*vir*) gene (regulator of *sxl* pre-mRNA splicing) which causes female-specific zygotic lethality in the embryonic stage and masculinizes the escapers^46^. Similarly, *D. melanogaster* mutant, *sans-filee* (*snf*^16^,^21^), a splicing regulator of *sxl* pre-mRNA, reduces the zygotic dose of Sxl to one copy number resulting into female-specific lethal maternal effect and a dominant masculinizing zygotic effect^47^. Consistent with this, we found the reduced amount of female-specific *Tctra* isoforms in masculinized genetic females developed from eggs laid by *Tctra* RNAi females, compared to control females (Fig. 5C). The escapers (genetic females) were converted to males that are fertile since virgin females mated with these males laid eggs that successfully developed into larvae. Detailed studies on the dosage compensation mechanisms in insects have only been done in dipterans^48^. Studies on the insects belonging to the orders Hymenoptera and Lepidoptera suggest the absence of global dosage compensation mechanism in these insects^49^,^50^. Further, the existence of dosage compensation in coleopterans is completely a black box since no study on this aspect has been performed in any insect belonging to this order. *Tctra* knockdown studies presented here, to our knowledge provides the first genetic evidence for the possible existence of dosage compensation in *T. castaneum*. Another likely explanation for female elimination from the *Tctra* RNAi progenies is an increase in non-disjunction in XX embryos, as seen in *D. melanogaster*^51^. In this insect, a partial reduction in *sxl* expression in the germline results in high levels of non-disjunction.

**Figure 6:**
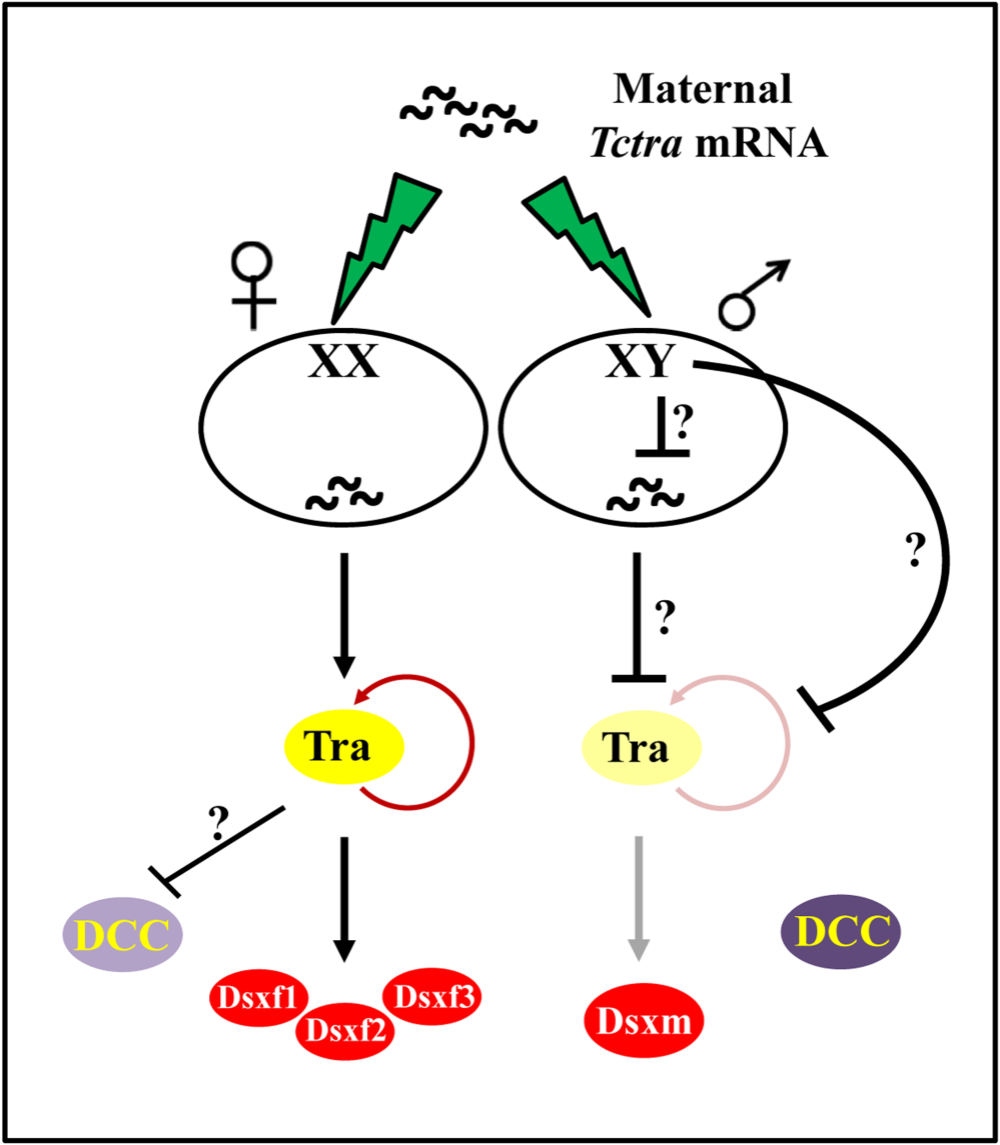
Model for sex determination in *T. castaneum*. Maternally transferred *Tc*Tra is translated to make *Tc*Tra protein only in females. This *Tc*Tra protein splices the zygotically transcribed *Tc*Tra pre-mRNA into female mode in turn production of *Tc*Tra protein. Continuous production of *Tc*Tra, in females, is ensured by the positive autoregulatory feedback loop. *Tc*Tra splices the *Tcdsx* pre-mRNA to produce three female-specific isoforms (*Tcdsxf1*, *Tcdsxf2* and *Tcdsxf3*). Also, *Tc*Tra inhibits some of the Dosage compensation components (DCC) to prevent the formation of active dosage compensation complex, in females. In males, an unknown dominant factor (M) inhibits the translation of maternally supplied *Tctra* transcript and/or degrades the maternal transcripts or inhibits its autoregulation. The lack of initial *Tc*Tra protein, in males, leads to the default splicing of *Tctra*, coding for truncated non-functional protein. In the absence of *Tc*Tra protein *Tcdsx* pre-mRNA splices in a male mode producing *Tcdsxm*. Absence of *Tc*Tra protein in males allows the formation of functional dosage compensation complex owing to the presence of all the DCC components (dark blue). Dark color represents active protein whereas corresponding light color represents truncated or inactive protein.

The nature of the inhibition of maternal *Tctra,* in males, is not known at this time. Though, different regulatory mechanisms are known to play role in the regulation of maternal mRNA^52^,^53^ we hypothesize the presence of a dominant male-determining gene (M factor) to be present on Y-chromosome of *T. castaneum* responsible for the inhibition of maternally transferred *Tctra* (Fig. 6). Similar prediction has been made for the male determination in *L. cuprina* also^29^. In *D. melanogaster*, *msl2* is the direct target of Sxl; the primary gene of *D. melanogaster* sex determination cascade. The observed defects in *Tctra* RNAi insects suggest that *msl2* (or some other dosage compensation component) may be direct or indirect target of *Tctra.* Also, RNAi studies on *Tctra* showed the requirement of *Tctra* throughout the life, in females, to maintain the splicing of *Tctra* and *Tcdsx* pre-mRNA in female mode. Data reported here provide the first steps towards understanding of sex determination pathway in a coleopteran insect which not only expands our understanding of sex determination mechanism in insects but also help in designing strategies for the control of harmful insect pests by employing parental RNAi of Tra/Fem and the sterile insect releases.

## Methods

### *Tribolium* *castaneum* strain, RNA isolation, PCR and RT-PCR

Young (0 day) Larvae, Pupae and adults of *T. castaneum* strain GA-1 were used in the experiments conducted. Pupa and adults were sexed based on the visualization of sex-specific structures. Dissected adults were frozen in liquid nitrogen and stored at −70°C until further use. Both RNA and DNA (simultaneously), from the same sample, was isolated using Trizol method (Invitrogen Corporation, USA). DNAse treated total RNA was denatured at 75°C for 5 min and immediately chilled on ice. First strand cDNA was synthesized with MMLV reverse transcriptase (Invitrogen, USA) using 17-mers polyT primer, according to the manufacturer’s instructions. Primers targeting male- and female-specific isoforms of *Tctra* were designed based on the sequence at the junctions of the first and second exons. PCR reaction conditions were as follows-Initial denaturation at 94°C for 2 min, 32 cycles of 94°C for 30 s, 60°C for 30 s, 72°C for 2 min and the final extension at 72°C for 10 min.

### Sequence analysis

Exons and introns, of *Tctra* were identified by aligning sequences of RT-PCR products with their corresponding genomic DNA sequences obtained from the Beetlebase and the NCBI. Exon-intron boundaries were confirmed by aligning the sequences through Spidey (http://www.ncbi.nlm.nih.gov/spidey/).

### Double stranded RNA (dsRNA) synthesis and injections

Using *T. castaneum* cDNA template and primers (shown in supplementary Table 2S) containing the T7 promoter sequence at their 5’ ends and sequence specific to common regions of *Tctra* was amplified by RT-PCR (Fig.1A). Purified amplicons were in-vitro transcribed to synthesize dsRNA using MEGAscript T7 kit (Ambion, Austin, TX). Amplicon from *Escherichia coli malE* gene was used to prepare control dsRNA. dsRNA injections were performed on the first day of final instar larvae, 0 h pupa or newly emerged male and female adults (~6 h PAE). The insects were either anesthetized with ether vapor (for larvae) or kept on ice (for pupa and adults) for 8–10 minutes prior to injections. dsRNAs (≈500–600 ng per insect) were injected on the dorsal side of larvae and pupae whereas on the ventral side in adults using an aspirator tube assembly (Sigma) fitted with 3.5″ glass capillary tube (Drummond) pulled by a needle puller (Model P-2000, Sutter Instrument Co.). Injected insects were allowed to recover for 8 h at room temperature (~22°C) and then transferred to standard conditions. Knockdown efficiencies of gene expression in the RNAi insects were calculated as the ratio of gene expression between target dsRNA injected and *malE* dsRNA injected beetles.

### Collection and staging of embryos

For the collection of unfertilized eggs, newly emerged virgin females were sex-separated and allowed to grow. After 5 days, the females were transferred to fine flour and eggs laid over 12 hr period were collected by filtering the flour through sieve of 250 µm pore size. For the collection of fertilized eggs, mated females were allowed to lay eggs in fine flour either for 1 hr or for 5 hr. Eggs collected during one hour period were frozen at 2 hr intervals until 12 hr after egg laying and stored at −70°C until further use. Thus, seven samples at 0–1, 2–3, 4–5, 6–7, 8–9, 10–11 and 12–13 hr after egg laying were tested. Similarly, eggs laid over 5 hr period were staged at 6–11, 18–23, 30–35, 42–47, 54–59, 66–71 hr after egg laying and hatched first instar larvae.

### Analysis of parental RNAi

To evaluate the effect of dsRNA mediated knockdown and depletion of *Tctra* from the early embryos in the next generation; newly emerged adults were sex-separated and allowed to grow for 5 days. dsRNA for *Tctra* or *malE* was injected to these adults and allowed to recover at room temperature for one day (24 hrs). Females and males were allowed to mate in a cup kept at standard rearing conditions. After 20 days, cups were checked for the number of larvae and the larvae were further reared to sex-separate them during pupal and adult stages.

### Quantitative real time PCR

Quantitative PCR was performed using the SYBR Green kit (Roche, USA) according to the manufacturer’s instructions. Female-specific reverse primer was designed based on the sequences at the junction of first and second exons (Fig. 1A). Male-specific forward primer was designed based on the sequence of male-specific exon (Fig. 1A). RNA isolation and RT-PCR was done as mentioned above. Three independent biological replicates were analyzed for each treatment. *Tribolium* *rp49* gene was used as an endogenous control to normalize the expression data and the gene expression level were analyzed by 2−ΔΔCt method^54^.

### Imaging and documentation

The gonads from the dissected insects were stained with acridine orange and the images were taken by Olympus 1×71 Inverted Research Microscope fitted with reflected fluorescence system. Acridine orange was excited using 502 nm laser line. “Megna Fire software” (version 1.5) was used to control the microscope, image acquisition and exportation of TIFF files. Figures of all micrographs were assembled using Adobe Photoshop element 9.

## Author Contributions

Both authors planned and conducted experiments and wrote manuscript.

## Supplementary Material

Supplementary Information Supplementary info

## Figures and Tables

**Table 1:** Effect of *Tctra* knockdown on the number of eggs laid/mating pair

Mating pair	Control female X Control male	knockdown female X knockdown male	Control female X knockdown male	knockdown female X Control male
**1**	70	0	63	0
**2**	65	0	68	0
**3**	68	0	65	0
**4**	72	0	70	0
**5**	67	0	68	0
**6**	68	0	71	0

**Table 2:** Effect of *Tctra* knockdown in the larvae on the development of sex-specific phenotypes in pupae and adults

				No of pupa		No of adults		adults survived 2 days PAE	
dsRNA injected	Larval instar	No of larvae injected	No of survivors	Female	Male	Female	Male	Female	Male
***malE***	4th	20	17	10	7	10	7	10	7
	5th	20	18	8	10	8	10	8	10
	6th	20	20	8	12	8	12	8	12
***Tctra***	4th	20	18	0	18	0	18	-	10
	5th	20	18	0	18	0	18	-	9
	6th	20	20	0	20	0	20	-	9

**Table 3:** Sex ratio of the progeny developed from the eggs laid by females injected with *Tctra* dsRNA

dsRNA	No of adults injected	No of survivors	No of progenies (larvae)	No of females (pupa or adults)	No of males (pupa or adults)
***malE***	5 female and 5 male	All	240	130	110
***Tctra***	5 female and 5 male	All	34	0	34 (31 genetic males + 3 genetic females)
