# Supplementary material for: Sex determination in beetles: Production of all male progeny by Parental RNAi knockdown of transformer
Source: Sci Rep. 2012 Aug 24;2:602. doi: 10.1038/srep00602 (PMC3426794; doi:10.1038/srep00602)
Supplement: Supplementary Information — Supplementary info [file srep00602-s1.pdf]

Title: Sex determination in beetles: Production of all male progeny by Parental RNAi knockdown of *tranformer*

Author names: Jayendra Nath Shukla and Subba R. Palli

Supplementary Material

References

[1] Ruiz, M.F. *et al.*, The gene transformer of anastrepha fruit flies (Diptera, tephritidae) and its evolution in insects. *PloS one* 2 (11), e1239 (2007).

[2] Pane, A., Salvemini, M., Delli Bovi, P., Polito, C., & Saccone, G., The transformer gene in Ceratitis capitata provides a genetic basis for selecting and remembering the sexual fate. *Development* 129 (15), 3715-3725 (2002).

[3] Lagos, D., Koukidou, M., Savakis, C., & Komitopoulou, K., The transformer gene in Bactrocera oleae: the genetic switch that determines its sex fate. *Insect molecular biology* 16 (2), 221-230 (2007).

[4] Concha, C. & Scott, M.J., Sexual development in Lucilia cuprina (Diptera, Calliphoridae) is controlled by the transformer gene. *Genetics* 182 (3), 785-798 (2009).

Figure Captions

Figure 1S Deduced amino acid sequence of TcTra with putative auto regulation domain (red), Arg/Ser domain (blue) and proline rich region (yellow) identified based on their similarity with those present in Tra/Fem homologs.

Figure 2S Conservation of putative Tra/Tra2 binding sequence. A) The consensus Tra/Tra2 binding sequences from *Drosophila melanogaster*, *Musca domestica*, *Anastrepha species* [1], *Ceratitis capitata* [2], *Bactrocera Oleae* [3] *and Lucilia cuprina* [4] are shown in boxes. B) Nucleotide identity in the genomic region of *Tctra* for ATCCA type and CAAT type putative Tra/Tra2 binding sequences. The overall height of each stack indicate the consensus sequences at that position (in bits) and the height of nucleotides represent the relative frequency of each nucleotide at that position.

Figure 3S *Tctra* genomic sequence (corresponding to the region spanned by female specific ORF) showing the presence of Multiple CAAT type (in green color) and ATCAA (in yellow color) type putative Tra/Tra-2 binding sites in the male specific exons and first intron. Four putative ISS sequences (underlined) and three putative RBP sites (in box) were also found. Common exons are shown in blue whereas male specific exons are in red. Black colored italics letters represents intron sequences. Intronic and exonic regions are indicated on the right side.

Table 1S: Number of eggs laid by virgin females mated with males developed from eggs laid by *Tctra* RNAi females

Mating pair	#matings	No of eggs laid/female	No of eggs hatched
Virgin females X Genetic males	31	50	all
Virgin females X Masculenized genetic females	3	40	all

Table 2S: Primers used

Primer name	Primer sequence
TradsRNAF	CGTGATTACCACTCACCGAGAC
TradsRNAR	GATACATCATCGGATGCCTCAA
PSIdsRNAF	TGTAAAATCCAAATGGCACCTG
PSIdsRNAR	TTCTCGGCAATCAAATCGTAGA
Tra-F	ATGTCGGGTTCAAAGAGCCAAG
Tra-R1	TCGTTCTGTCCTTCACTCTCACAC
Tra-R2	GAGATTTACACGGACGTCTTCG
Tra-female-specific-R	GCGGACGCCTTCATCAGGT
Tra-male-specific-F	AGCGCTACGACGTAACAATTTG
PSI-qRTF	GAAGAAAGGTCCAAGCACCAGT
PSI-qRTR	CCGACTGAAGTCTCGTGATCTG

Fig. 1S

MSGSKSQGTPEVLMQLDKNELQSIKVKITRRNVNKPYNTALLQRGLEDPADLKVHLR PDEGVRPIFDREDVRVNLRFSDSPPVNPPKPGNDKARSRSPARHRSPQPHTSRDYPRRR DYHSPRHRRSSSRSKKVVYKTSPPRYRRSRSPRRSRDRSPRDRYRRRTRSPSPYRRSRY SRSPDYRRYDRRSRSPKRHRYTPERDRHEIAYQPAGIPPYLQGPEMWIPAPRIPPMIPP MAFPYPPLRHPMMYRGPPFRPRIIYNNPRPRVTQAIASATVTTTTTTATPEVTECESEG QNDNNVTEETTDENK

Fig. 2S

A)

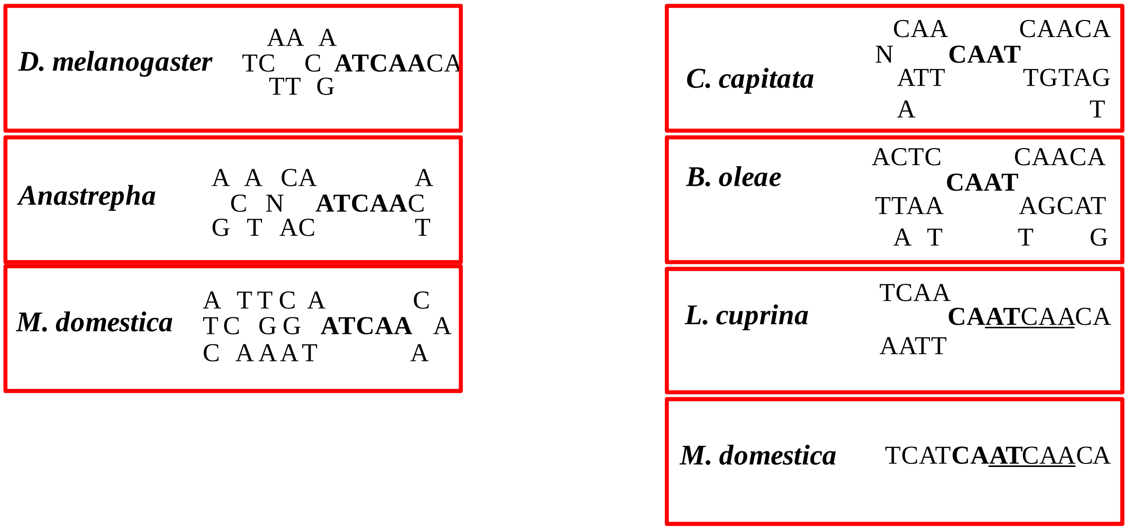

B)

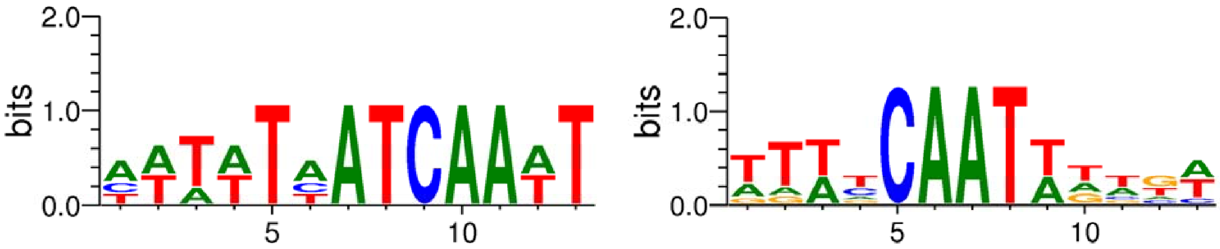

Fig.3S

Common Exon 1

ATGTCGGGTTCAAAGAGCCAAGGAACACCCGAAGTTCTAATGCAGTTGGACAAAAATGAGCTGCA
AAGCATCAAAGTGAAGATTACTAGAAGAAATGTTAACAAGCCGTATAATACGGCCCTTCTTCAGC
GGGGTTTGGAGGACCCCGCTGACCTTAAAGTTCATTTAAGACCTGGTAATTGGTTTTCATAGTCT

Male specific exons

TGACGAAGTTTGCTCTACTTCTGTAGTTTCTCCGATTGCAATAATTATTACAGGTTTTTGTAATT
ATTTTTATTTAGGATAATAAAGAAGAACACAAATTTTGAAGACACTTGACTACGACAAAGGCCAG
CGCTACGACGTAACAATTTGCATAAAAACAGTCAGAAAATTTCATACAAAAATGTAAGTTCTTTC

Intron

TAGATTAATAAATTATCTATAATTAGTTTTTTCCTGATTAATTTAATTTAATTTTCTAACGTCAC
GTCCATAAATGCTTATTTGATGCTGAAGTTTTCATGTTTTTCAATAGTGCATGTTGCAAGGATCA
AGCAAGAAGACGGAGGAATTGAAGAAGAATTGTTATAAAAAAAATTATCAATTTAGTTTGACATT
ATAATCAAATAACTTTACATTGCATGTACGTAAAATTTTATTTGTTTTGAGTAAAAGTCATTCAT
TAAAATTAACTTAAGTTGCATGCCATTATTTTGAGTAATTTTTATTATTTTGTAAATTTCTTTAA
ATTGGAAGAAAATGGAGAAGAAAGAAGAGCAGAAGACCCAAGAAGCCATTGTTTGGAAGACTTGT
TATTTGTCCAATTGTTTTTGTAGCAGTAAAATTTTTTAATCAATTTAAATTATTACGAAGAATCG
TAACATTAATAATAATTTTTTTGTTCATTTAAATTATTCTAAAAAGTCGCTTCAAGTTTTTTAATA
ATTTCACTGATTATTCCTATTAATTTTTTTTCGTAAATTTAAATTTCTTTAAACTGGGGGAAGATG
AACGAAGAATGAAGACACGGAGCCGAACCTGAAGAATTTTATTTATGTCAATAATTGTTAATTTA
ATTCGTTCGAAAAAGTCATTTCATCAAATGAGAGAACCGCTTGATTTCAGATGAAGGCGTCCGCC

Common Exon F (2) and M (3 or 4)

CCATTTTTGACCGCGAAGACGTCCGTGTAAATCTCAGATTTAGTGATAGCCCCCCTGTAAATCCC
CCAAAACCCGGAAACGACAAAGCCAGGAGTCGAAGTCCGGCGAGACATCGCAGTCCCCAGCCCCA
CACCTCCCGGGATTACCCCAGACGTCGTGATTACCACTCACCGAGACATCGGTACTTCCCCCAAA

Intron

CAAACCACAAAAATTCCAATAACGTAATTTCAGACGTTACAGACGTAGCAGATCGCCGAGGCGAA

Common Exon F (3) and M (4 or 5)

GCCGCGACAGGTCCCCCAGAGACAGGTACCGGAGGCGTACTCGGAGCCCCTCCCCCTATCGCCGG
TCCCGGTATAGTAGATCGCCTGATTACCGACGGTATGACCGGCGGAGTCGTTCCCCCAAAAGGCA
CAGATACACTCCCGAGAGAGATAGACATGAAATCGCGTACCAACCGGCGGGGATTCCCCCCTATT
TACAAGTGAGTGGTTTTAGGTTTGTTGGGTTGGTGGTAATGGTTTTACAGGGGCCTGAAATGTGG

Intron

ATACCTGCTCCGAGGATTCCACCGATGATACCGCCGATGGCTTTTCCGTACCCCCCTTTGAGGCA
TCCGATGATGTATCGGGGGCCCCCTTTCAGGCCTAGGATTATTTATAATAATCCCAGACCTAGAG
TGACGCAAGCCATTGCAAGTGCGACTGTTACAACAACCACGACAACGGCAACGCCTGAAGTTACG
GAGTGTGAGAGTGAAGGACAGAACGATAATAATGTAACTGAAGAAACGACTGATGAAAATAAATA
G

Common Exon F (4) and M (5 or 6)
